# Ecological and evolutionary implications of genomic structural variations

**DOI:** 10.3389/fgene.2014.00326

**Published:** 2014-09-16

**Authors:** Frédéric J. J. Chain, Philine G. D. Feulner

**Affiliations:** Department of Evolutionary Ecology, Max Planck Institute for Evolutionary BiologyPlön, Germany

**Keywords:** structural variation, SV, copy number variation, CNV, inversions, ecological genetics, evolutionary genomics, genome evolution

Large genomic segments spanning millions of nucleotides commonly differ between any two genomes, including between monozygotic twins (Bruder et al., 2008). These structural variations (SVs) include deletions, insertions, duplications, inversions, and translocations. SVs have been associated with human genetic diseases (Weischenfeldt et al., 2013), but can also facilitate adaptation (Iskow et al., 2012) and speciation (Noor et al., 2001; Rieseberg, 2001). In this research topic, the contributed articles offer insights into the ecological and evolutionary implications of genomic SVs, emphasizing the advances, limitations, and importance of studying the evolution of structural polymorphisms in model and non-model organisms.

The recent developments in genomic technologies and methodologies allow the study of SVs in basically any organism, including ecological models with limited prior genetic information available. In this research topic, Fan and Meyer (2014) provide an extensive catalog of various types of genomic variation across four recently diverged cichlid lineages and speculate on the relevance of SVs for one of the largest adaptive radiations in vertebrates. The phylogenetic context of their study suggests that point mutations are commonly obtained at the basal nodes, whereas the rates for acquiring SVs are increased at the tips of their phylogeny. This study provides a starting point to examine the role of SVs in the diversification and speciation of cichlids.

Inversions are SVs that may be particularly effective in promoting speciation due to a subsequent reduction in recombination when heterozygous (Butlin, 2005). Feder et al. (2014) summarize previous theoretical efforts evaluating the impact of inversions on speciation, and assess the consequences of inversions in the divergence of two populations through simulations. The authors examine how the genomes of these populations become distinct through recombination barriers. Results from their simulations suggest that conditions most favorable to incite speciation involve inversions that are already fixed between populations before secondary contact.

Segregating inversions occur in a variety of systems (Faria and Navarro, 2010) including the mosquito *Anopheles*. Ayala et al. (2014) review the relationship between inversions and adaptive traits in *Anopheles*. Several inversions across eight species have been linked to phenotypic traits including insecticide resistance, higher tolerance to xeric environments, and mate choice. The authors urge that further investigations on the adaptive effects of inversions in *Anopheles* are needed to reveal causal mechanisms, while providing valuable information on regulating the spread and behavior of this important vector of human disease.

Offering a non-eukaryotic perspective of SVs, López-Pérez et al. (2014) report on the role of structural genomic polymorphisms across a variety of aquatic microbial species. The authors investigated the prevalence of genetic exchange of “flexible genomic islands” between strains in several species. These islands are of different sizes and consist of different genes, however the authors suggest their exchange occurs too infrequently to be an important short-term strategy for niche establishment, but rather may be involved in modulating phage-sensitivity.

Copy-number variations (CNVs) are a prevalent type of SV that make up an extensive portion of genetic diversity. Katju and Bergthorsson (2013) contribute a review on the mutation rate and fitness consequences of CNVs, contextualizing CNVs in the rich history of duplicate gene evolution research. The authors provide the impetus for studying population-level duplications and deletions, their adaptive potential, and their mutation rates, summarizing that CNVs occur more frequently than SNPs based on mutation accumulation lines. While the majority of newly arisen mutations are deleterious and soon eliminated by purifying selection, the rate of duplication may have a large impact on the evolution of duplicated genes and organismal fitness.

The divergence of CNVs between populations may help identify candidate regions under selection. Bryk and Tautz (2014) tested the association between CNVs and expression, as well as the differentiation of CNVs between recently diverged natural mouse populations. No association between CNVs and gene expression was found, and CNVs that were differentiated between the populations were mostly located in intronic or intergenic regions. The authors did however find evidence for selective sweeps around some differentiated CNVs using microsatellite length heterozygosity, hinting at a potential adaptive role that might be worth pursuing in future studies.

The evolutionary and functional consequences of CNVs in livestock are an important area of research with economic relevance and are reviewed by Bickhart and Liu (2014). The authors list mechanisms of SV formation, and present SVs associated with distinct phenotypic traits in domesticated animals. These include artificially selected traits such as coat color, and other variants relevant to agricultural productivity. The authors indicate a need for progress on SV detection methods and improvement of reference genomes and annotations, which would help in expanding what is already known about SVs in these species.

Focusing on the evolutionary implications of SVs, Keane et al. (2014) review studies of SVs in mice and propose further research directions in this field. SV detection methods are first summarized, followed by evidence for the functional importance of SVs in mice and the role of transposable elements in genome evolution. The authors discuss methods to access previously published data of SVs in mice, but point out current limitations of the existing approaches to analyze these data. As it stands, it is difficult to compare SVs across studies, especially when different technologies and methods of detection are utilized.

Given that SVs are important to consider when studying genetic diversity and genome evolution, as highlighted by the contributions to this research topic, improvements in SV detection and analysis should be a priority to better evaluate the impact of SVs. Most current methods are poor at defining breakpoints at a fine scale, making it difficult to determine the mechanism of SV formation—an essential requirement for understanding the evolution of SVs. Moreover, the ability to accurately genotype SVs would allow a population genetic framework analysis that can make use of allele frequency changes to determine the evolutionary dynamics of SVs. Despite these limitations, essentially any organism can now be screened for SVs, which will lead to increasing our knowledge on the ecological and evolutionary implications of genomic SVs.

## Conflict of interest statement

The authors declare that the research was conducted in the absence of any commercial or financial relationships that could be construed as a potential conflict of interest.
